# Mature cystic teratoma of the ovary with a grossly visible, completely developed intestinal loop: A case report and review of the literature

**DOI:** 10.1097/MD.0000000000034081

**Published:** 2023-06-30

**Authors:** Yuanyuan Cao, Bihui Wang, A-Ran Jia, Xuejian Li, Liuqing Yang, Zanhui Jia

**Affiliations:** a Second Hospital of Jilin University, Jilin University, Changchun, Jilin Province, China; b The First Affiliated Hospital of Xinjiang Medical University, Xinjiang Medical University, Xinjiang, China.

**Keywords:** gastrointestinal tract, malignant transformation, mature cystic teratoma

## Abstract

The mature cystic teratoma of the ovary (MCTO), is composed of mature differentiated elements, thus showing highly differentiated tissue and highly morphological heterogeneity. Although gastrointestinal epithelium can be identified in 7% to 13% of cases of MCTO, the occurrence of visible, functional, and fully developed loop tissue is rare in clinical practice.

**Patient concerns:**

A 17-year-old female patient presented with persistent abdominal pain.

**Diagnosis:**

The patient was diagnosed with MCTO where a visible, functional intestinal loop was observed during laparoscopic surgery. Microscopy of the intestinal structure indicated a well-organized, intact layer of intestinal wall.

**Interventions:**

An emergency single-port laparoscopic excision of the right ovarian cyst and histopathology were performed.

**Outcomes:**

After 2 years of follow-up, there were no signs of recurrence in the patient.

**Lessons:**

The immune signature of CK7−/CK20+ is characteristic of tumors of gastrointestinal origin and can be used to distinguish tumors associated with mature cystic teratoma. Besides, gynecologists should keep an eye on the possibility of malignant transformation malignant transformation of MCTO.

## 1. Introduction

The term “teratoma” refers to a neoplasm made up of somatic cell populations differentiated from germ cell populations in the ectoderm, endoderm and mesoderm.^[1]^ The degree to which it is benign or malignant depends on the maturity of the tumor tissue. Among all teratomas, the mature cystic teratoma of the ovary (MCTO) is the most common benign germ cell neoplasm of the ovary and has an incidence of 14.2 cases per 100,000 people yearly.^[2]^ MCTO most frequently occurs in women of reproductive age.^[3]^ MCTO is a slowly growing tumor, and its long-term recurrence rate after surgery is 4.7%.^[4]^ Hence, fertility - sparing surgery has been performed on adolescent and reproductive female with MCTO.^[3]^

Histologically, 7% to 13% of MCTO cases include intestinal epithelium.^[5]^ Previously, gastrointestinal wall or epithelium within the MTCO that was identified at the microscopic level has been reported. Herein, we report the first case report of the formation of a grossly visible, completely developed and peristaltic gastrointestinal tract in the MCTO.

## 2. Case report

**Figure 1.**
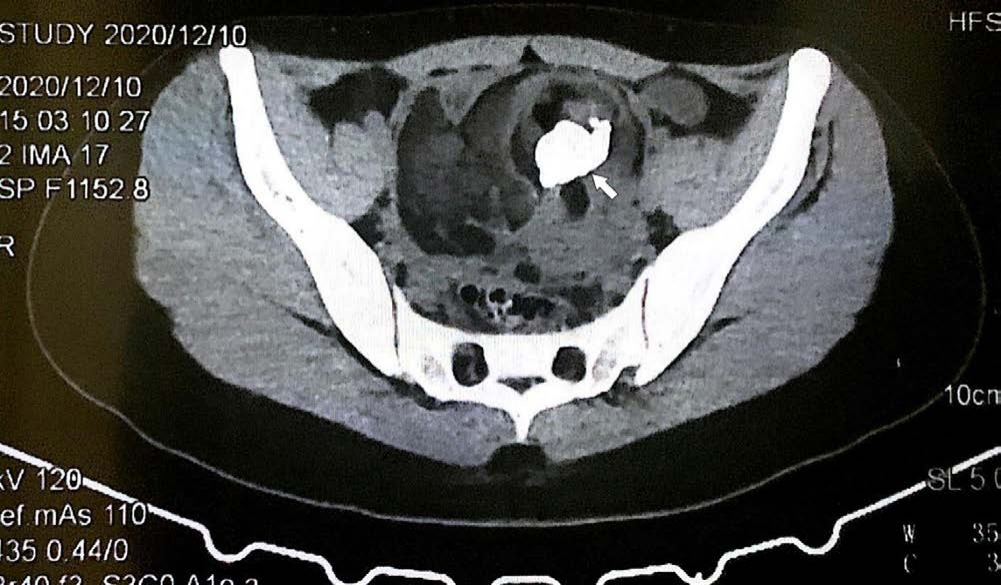
Preoperative abdominal CT images of the patient, the white area indicated by the white arrow is the lesion location, where fat density and high-density shadows were seen.

A 17-year-old girl, with persistent pain in her right lower abdomen for 2 days, was admitted to the emergency department of the Second Hospital of Jilin University (our hospital) on December 11, 2020.The patient is an adolescent female, no sexual intercourse, with regular menstruation, 13 4-5/28, moderate menses volume, dysmenorrhea (−), blood clots (−), LMP on October 30, 2020. On December 10, 2020, the patient noted dull lingering pain in the right lower abdomen without obvious cause. On the same day, she went to a local hospital for an abdominal ultrasound scan, which demonstrated a moderate echogenic cluster measuring approximately 13.0 × 7.8 × 12.0 cm, which was considered to be a teratoma, with uneven internal echogenicity: a small amount of anechoic and patchy strong echogenic accompaniment and a mass of moderate echogenicity was seen. The following day, the abdominal CT was performed, which revealed that a mixed density shadows were seen in front of the uterus, and fat density and high-density shadows were seen inside this mass (Fig. 1). So it was suggested to transfer to the tertiary level hospital for emergent care. That same night, she was admitted to our hospital with the initial diagnosis of a “pelvic mass (ovarian tumor pedicle torsion?).” No other positive symptoms were noted during the course of the disease. Laboratory evaluation demonstrated normal blood counts, serum electrolytes, liver and kidney function tests, carcinoembryonic antigen, alpha fetoprotein, CA19-9, HCG, and human epididymal protein 4, however, the tumor markers CA125 was 40.8 U/mL (normal value is under 200 U/mL in pre menopausal women), and sugar antigen CA72-4 was 10.70 U/mL (0–6.9 U/mL).

**Figure 2.**
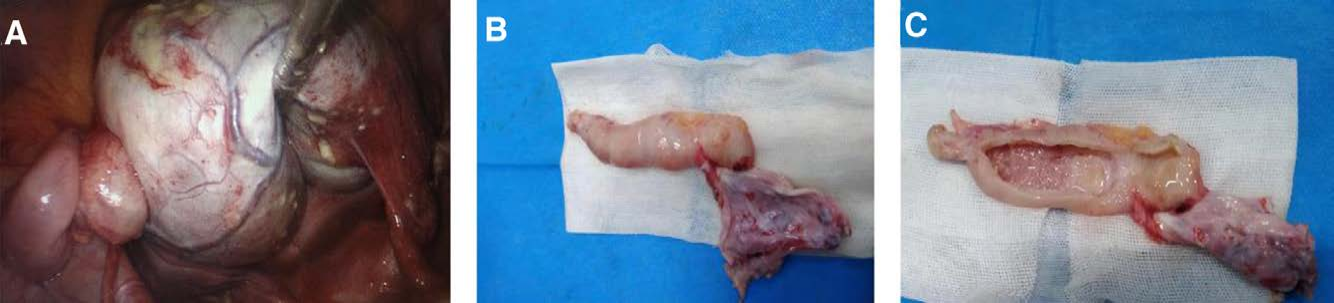
(A) Mature cystic teratoma of the right ovary and intestinal loop tissue developed by ovarian tumor were seen under laparoscopy. (B) A gross view of the intestine loop developed by mature cystic teratoma. (C) The intestinal canal in a mature cystic teratoma of the ovary was dissected to reveal clear fluid flow, while intestinal mucosal tissue was visible in the canal.

An emergency single-port laparoscopic excision of the right ovarian cyst was performed at 02:15 On December 12, 2020. Intraoperatively, there was a cystic mass measuring approximately 13.0 × 8.0 × 12.0 cm, white in color, located within the right ovary. Furthermore, exploration of the tumor revealed curved, a fully developed and peristaltic intestinal loop of 7.0 × 2.0 × 2.0 cm in size were attached to the left wall of the ovarian tumor, unrelated to normal surrounding bowel tissue (Fig. 2A). We decided to perform a right ovarian tumor debulking procedure. A 1 cm incision was made on the surface of the right ovarian tumor. The fat, hair and bone in the teratoma were visible to the naked eye. After the tumor had shrunk, the tumor and the loop of the intestine were completely removed from the right ovary. During the excision, the bowel was seen to move slowly until it did not move. The cyst was placed in a sterile specimen bag, clamped to the outside of the sterile bag and removed from the umbilical cord. There were no notable abnormalities in other organs of the pelvis and the abdominal cavity.

Observations of the right resected ovarian cyst demonstrated the surface of this intestine was smooth with a folded root surface, and a greater omentum, which was extremely rich in blood, was connected with the lower part of the intestinal canal (Fig. 2B). After the soft intestinal canal was dissected, a small amount of clear yellowish fluid was seen following from the intestine, and the bowel mucosa was visible on the inner bowel wall (Fig. 2C). Frozen pathologic sections were not performed due to nighttime emergency surgery.

**Figure 3.**
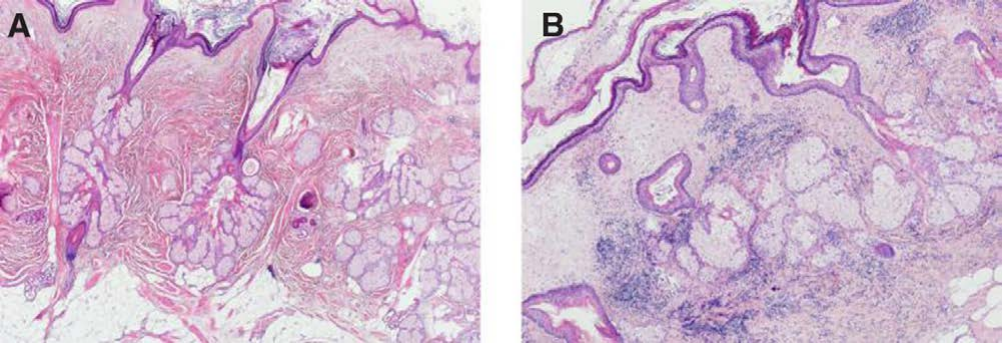
Microscopic image of mature cystic teratoma of right ovary (A: HE × 20; B: HE × 80).

**Figure 4.**
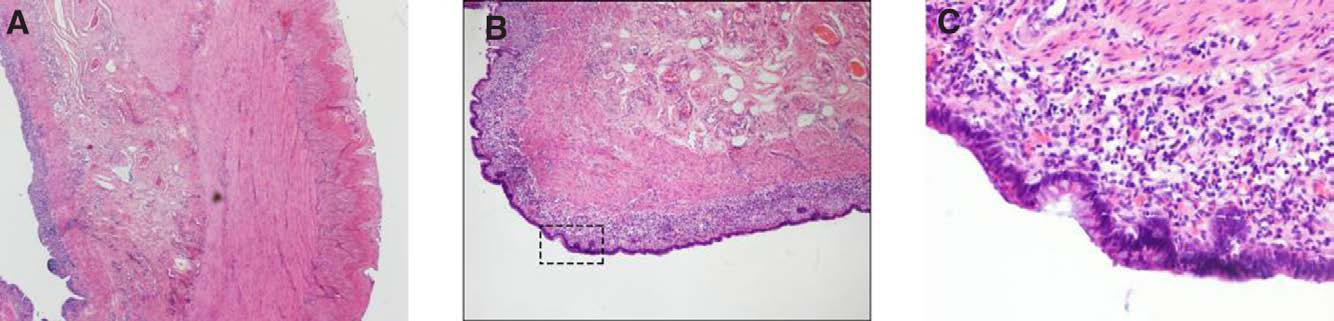
Microscopic image of intestinal tissue of ovarian mature cystic teratoma growth (A: HE × 20; B: HE × 80; C: HE × 200).

The pathologic diagnosis of the ovary was a mature cystic teratoma with hyperplasia of the localized lymphoid tissue. Microscopic sections of the mature cystic teratoma included common elements of skin, hair follicles, adipose tissue (Fig. 3). The sections from the grossly identifiable intestinal loop indicated intact layers of intestinal wall with mucous, submucous, muscularis, and serous layers (Fig. 4). However, the muscular layer was thicker and the mucosal layer was thinner compared to the normal intestinal collaterals tissue. Immunohistochemistry stains indicated that CD2(+), CD3(+), CD20(partial +), Pax-5(partial +), CD43(+), CD23(scattered +), CD21(focal +), CyclinD1(−), Bcl-2(+), CD10(−), Ki67(positive rate 1%), CK (AE1/AE3) (−).Postoperative diagnosis: mature cystic teratoma of the right ovary.

After the operation, the patient was discharged on the second postoperative day. After 2 years of follow-up, she remains free of disease.

## 3. Discussion

The MCTO is composed of variable, well-differentiated derivatives of the 3 germ cell layers, of which the ectoderm is the most prominent.^[5]^ In MCTOs, visual findings of teeth, bones, cartilage and brain tissue are frequently observed, but the gastrointestinal epithelium was found in only 7% to 13% of cases.^[5,6]^ Table 1 is a review of the organized gastrointestinal development in MCTO in the literature to date. Woodfield reported the first case of a nearly fully developed gastrointestinal tract, which histologically represents the entire gastrointestinal tract from esophagus to colon in a benign cystic teratoma and reviewed 15 cases of the organized gastrointestinal development dating back to 1876.^[6]^ Although Gastrointestinal-type epithelium is occasionally observed in MCTO, the existence of intact intestinal wall in this tumor, including the muscular layers, is very rare.^[7]^ Fujiwara et al reported in 1995 2 cases of MCTO with intact segments of the intestinal wall microscopically. The pathological type of 1 case was benign appendiceal mucinous cystadenoma, and the other was intestinal-type adenocarcinoma, including complete colonic structures and bronchial epithelium.^[8]^ Ping Tan et al in 2003 reported a case of mature cystic teratoma with histologically complete colonic wall structure, tubular mucinous cystadenoma structure, and transition zone structure in between. The authors showed that mucinous cystadenoma originated from the colonic epithelium of mature cystic teratoma.^[7]^ In 2016, it was also reported that mature cystic teratoma had complete colonic structure.^[4]^ In each of the above cases, the gastrointestinal wall structure was only found under a microscope. Mi Jung Kwon reported the first case of clearly visible, well-organized bowel loops of intestine and mesentery in mature cystic teratoma of malignant mixed germ cell tumor combined with a yolk sac tumor and a mature cystic teratoma in the same ovary.^[5]^ However, no active intestinal tissue was found in all cases of MCTO. In this case, completely developed and active intestinal loop in MCTO was firstly noted grossly and microscopically.

**Table 1:** Organized gastrointestinal development in benign cystic teratomas.

Histology	Tumor size (cm)	Symptom	Feature	Tumor marker	Treatment	Author
MCTO with intestinal-type tissues	5.0	Pain in the lower abdomen	Closely resembling intestine, coiled on itself, attached to the wall of the cyst by a structure resembling mesentery	-	Bilateral cystectomy	Andrews HR (1912)^[16]^
	17.0 × 12.5	Intermittent pain in the right flank which radiated posteriorly.	Containing extra cystic colon with mesentery and meconium	-	RSO	Bernstine et al (1959)^[17]^
	9.0 × 6.0 × 5.0	Dull right lower quadrant pain, bloating, and vague abdominal complaints.	With almost complete development of the gastrointestinal tract (from esophagus to colon)	-	RSO	Woodfield at al (1985) ^[6]^
	5.0 × 5.6	-	With complete colon structure	-	Laparoscopic LSO	Ki et al (2016)^[4]^
MCTO with intestinal-type secondary benign neoplasms	5.0 × 7.0	A pelvic pain	With formation of complete segments of intestinal wall, containing a benign mucinous cystadenoma of the appendiceal type	-	Laparoscopic RSO	Fujiwara et al (1995)^[8]^
	18.0 × 12.0 × 9.0	A recurrent yeast infection	Containing a complete colonic wall in continuity with an endocervical-type mucinous cystadenoma	CA125:89 U/mL	LSO	Tang P et al (2003)^[9]^
	9.5 × 6.5 × 4.0	Complaints of right lower abdominal pain.	With intestinal structures harboring intestinal-type mucinous neoplasm, mimicking low-grade appendiceal mucinous cystadenoma	CEA:34.9 ng/mL	TAH + BSO	Maki Takao et al (2018)^[15]^

BSO = bilateral salpingo-oophorectomy, CEA = carcinoembryonic antigen, LSO = left salpingo-oophorectomy, MCTO = the mature cystic teratoma of the ovary, RSO = right salpingo-oophorectomy, TAH = total abdominal hysterectomy.

Most MCTOs are asymptomatic, growing slowly at a rate of 1.8 mm per year, unless complications or paraneoplastic syndromes develope.^[9]^ In this case, there was no obvious cause of the pain in the lower right abdomen, presumably related to bowel cramping pain caused by a lack of space for intestinal movement in the MCTO. The normal gastrointestinal tract is under the joint control of the central nervous system, the autonomic nervous system and the enteric nervous system. The enteric nervous system is known as the “second brain” and consists of 2 plexuses. One is the interosseous plexus, located between the longitudinal and circumferential muscular layers of the intestinal wall, which provides innervation to both muscle layers to control peristalsis. The other is the submucosal plexus, located under the mucosal epithelium, which could sense the intraluminal environment, regulate blood flow in the gastrointestinal tract and control the function of epithelial cells.^[10]^ The pathological findings of the cases reported by Kwon et al^[5]^ suggest that interosseous plexus was present between the inner and outer muscle layers of the intestinal wall in the intestinal collaterals tissue in the ovarian tumor. However, the intestinal collaterals tissue in this case was not active and no intestinal fluid tissue was reported to date. In contrast, we grossly recognized the peristaltic intestinal canal in the MCTO, and microscopically confirmed an intact intestinal structure characterized by a thickly developed muscular layer and a poorly developed mucosal layer. The intestinal canal was dissected and about 10 mL of clear yellowish intestinal fluid was found, which showed that this intestinal canal has a reasonably well-developed nervous system.

Malignant transformation occurred in approximately 2% of all MCTO cases, arising from any of the constituent tissues of the teratoma; Among malignant transformations, squamous cell carcinoma has been reported to occur in approximately 80% of cases, and adenocarcinoma account for 6.8%.^[11]^Gastrointestinal adenocarcinoma arising in MCTO is extremely rare.^[12]^It is challenging to tell whether an MCTO has undergone a malignant change prior to surgery. However, a malignant transformation can be highly suggested in association with advanced age (>40 years), large tumors (>10 cm) or elevated serum tumor markers, or an uneven border on a CT scan that forms an obtuse angle with the inner cyst wall. Therefore, pathologic examination by frozen sections is necessary.^[12]^ However, we were unable to frozen section pathological examination, because the patient underwent an emergency operation at night.

The surgical stage and tumor histology determine the current course of treatment for mature cystic teratomas that have undergone malignant transformation.^[12]^ Ovarian mature teratoma originates from germ cells, but its abominations occur in somatic cells of teratoma. Therefore, Den Bakker et al^[13]^ suggest that adjuvant chemotherapy for patients with malignant transformation of MCTO cannot simply be transferred to chemotherapy regimens for malignant germ cell tumors and should be targeted according to the pathologically distinct sources of the sinister components. To determine the histological type of secondary malignancy in MCTO, a combination of histology and immunophenotype is required. A CK20 positive/CK7 negative pattern has been reported to be characteristic of tumors of gastrointestinal origin.^[3]^ Gastrointestinal adenocarcinomas secondary to MCTO most often exhibit diffuse expression of CK20 as well as negative expression of CK7.^[14]^ It has also been documented that CDX-2, choriocapillaris and CK-20 were strongly positive and CK-7 was negative as its characteristic.^[15]^

## 4. Conclusions

In this case, it is extremely rare for MCTO to develop a visible, fully developed and active intestinal loop tissue, which has excellent peristalsis function and secreted intestinal fluid in the intestinal tube. In fact, it expands the data for the cases of MCTO. At the same time, the intestinal tissue of mature cystic teratoma of ovary may become cancerous, so the discreetly follow-up of patients should be emphasized. However, the question to be resolved is whether histological shifts in MCTOs should influence chemotherapy options. Ultimately, further accumulation of clinical case evidence data is needed to find a more mature diagnostic and treatment plan.

## Acknowledgments

We have obtained a signed consent form from the patient and her guardian and this form will be filed with my records. We would like to thank the patient for their participation in this study.

## Author contributions

**Conceptualization:** Bihui Wang, Zanhui Jia.

**Data curation:** Yuanyuan Cao, A-Ran Jia, Liuqing Yang.

**Formal analysis:** Yuanyuan Cao, A-Ran Jia, Zanhui Jia.

**Investigation:** Xuejian Li, Zanhui Jia.

**Methodology:** Yuanyuan Cao, Xuejian Li.

**Project administration:** Bihui Wang, Zanhui Jia.

**Resources:** Liuqing Yang.

**Software:** Bihui Wang.

**Supervision:** Zanhui Jia.

**Writing – original draft:** Yuanyuan Cao.

**Writing – review & editing:** Bihui Wang, Zanhui Jia.
